# Exploratory Assessment of Short-Term Antecedent Modeled Flow Memory in Shaping Macroinvertebrate Diversity: Integrating Satellite-Derived Precipitation and Rainfall-Runoff Modeling in a Remote Andean Micro-Catchment

**DOI:** 10.3390/biology15030257

**Published:** 2026-01-30

**Authors:** Gonzalo Sotomayor, Raúl F. Vázquez, Marie Anne Eurie Forio, Henrietta Hampel, Bolívar Erazo, Peter L. M. Goethals

**Affiliations:** 1 Facultad de Ingeniería, Universidad de los Hemisferios, Paseo de la Universidad, 300, Quito 170147, Pichincha, Ecuador; 2 Departamento de Ingeniería Civil, Facultad de Ingeniería, Universidad de Cuenca, Av. 12 de Abril S/N, Cuenca 010101, Azuay, Ecuador; raulfvazquezz@yahoo.co.uk; 3 Laboratorio de Ecología Acuática (LEA), Facultad de Ciencias Químicas, Universidad de Cuenca, Víctor Manuel Albornoz S/N y Av. de los Cerezos, Cuenca 010215, Azuay, Ecuador; hennihampel@gmail.com; 4 Department of Animal Sciences and Aquatic Ecology, Faculty of Bioscience Engineering, Ghent University, Coupure Links 653, 9000 Ghent, Belgium; marie.forio@ugent.be (M.A.E.F.); peter.goethals@ugent.be (P.L.M.G.); 5 Instituto Nacional de Meteorología e Hidrología (INAMHI), Quito 170517, Pichincha, Ecuador; bolivar.erazo@gmail.com; 6 Departamento de Gestión de Recursos Hídricos, Empresa Pública Metropolitana de Agua Potable y Saneamiento de Quito, EPMAPS Agua de Quito, Quito 170519, Pichincha, Ecuador

**Keywords:** ungauged catchments, satellite-derived rainfall, rainfall–runoff modeling, hydrological memory, benthic macroinvertebrates

## Abstract

Estimating runoff in ungauged catchments remains a major challenge in hydrology, particularly in remote Andean headwaters where limited accessibility and budgetary constraints hinder the long-term operation of monitoring networks. This study integrates satellite-derived rainfall data, hydrological modeling, and benthic macroinvertebrate diversity analysis to explore how short-term antecedent flow conditions relate to temporal variation in community structure. The research was conducted in a pristine 0.26 km^2^ micro-catchment of the upper Collay basin (southern Ecuador). Daily simulated discharge was used to compute antecedent flow descriptors representing short-term variability and cumulative changes in stream conditions, which were related to taxonomic (i.e., H = Shannon diversity, E = Pielou evenness, and D = Simpson dominance) and functional indices (i.e., Rao = Rao’s quadratic entropy, FAD1 = Functional Attribute Diversity, and wFDc = weighted functional dendrogram-based diversity) using Generalized Additive Models. Results showed progressively higher hydrology–biology associations with increasing antecedent flow integration length, suggesting that biological variability responds more strongly to cumulative than to instantaneous flow conditions. Among hydrological descriptors, the cumulative magnitude of negative flow changes was consistently associated with taxonomic diversity. H and E showed more coherent and robust patterns than functional metrics, indicating a faster response of community composition to short-term hydrological variability, whereas functional diversity integrates slower ecological processes. While based on modeled discharge under severe hydrometeorological data limitations, this study provides a practical ecohydrological starting point for identifying short-term hydrological memory signals potentially relevant to aquatic biodiversity in ungauged headwater systems.

## Simple Summary

Studying river flow effects on aquatic ecosystems is challenging in isolated mountain regions where sustained water data are lacking. Small Andean headwater streams are a prime example: they are crucial for biodiversity but are seldom studied because of their remote locations. This study integrates satellite rainfall data, hydrological models, and field observations of aquatic insects to investigate how short-term streamflow fluctuations influence biological communities. We focused on a small, pristine mountain catchment in southern Ecuador and analyzed how recent flow conditions over days to weeks were related to changes in the diversity of aquatic organisms. Our results show biological communities respond more strongly to accumulated flow conditions over time than to single flow events. We also found that traditional measures of species diversity reacted more quickly to changes in flow than functional characteristics of the community, which reflect slower ecological processes. By using modeled streamflow, this study shows a practical and transferable approach for investigating flow–ecology relationships in rivers where direct monitoring is not available. The findings are valuable for improving ecological assessments, conservation planning, and water management in remote freshwater systems where monitoring data are scarce.

## 1. Introduction

Flow variability is widely recognized as a primary driver of aquatic community structure and function, as changes in discharge regulate habitat stability, disturbance frequency, and resource availability [1,2,3]. Because hydrological fluctuations directly shape ecological responses, understanding freshwater ecosystems increasingly requires a joint perspective that integrates hydrological processes with biological dynamics. This conceptual convergence has led to the development of ecohydrology, an interdisciplinary framework that links physicochemical stressors with biological receptors to improve ecosystem understanding and support sustainable water-resource management [4,5]. Several studies identify water flow as a primary regulator of population dynamics, taxonomic composition [6], and functional traits of benthic macroinvertebrates, which are among the most widely used bioindicators in freshwater monitoring [7,8,9,10]. Flow variability influences not only hydraulic conditions but also habitat stability, resource availability, and the disturbance-recovery dynamics that determine community assembly and resilience in running waters. These patterns are relevant in tropical lotic systems, where flow pulses are markedly differentiated between rainy and dry seasons [11]. Despite the scientific relevance of such findings (e.g., for environmental flow regulation), quantifying ecohydrological relationships between water quantity and aquatic communities (such as benthic macroinvertebrates) remains a challenge, particularly in remote areas where flow gauge stations are difficult to implement and/or manage. One way to overcome this limitation is by applying rainfall–runoff modeling [12], since rainfall is relatively easier to measure and maintain through simple rain gauges, even in isolated regions. Yet, in Ecuador’s high-Andean regions where the Páramo ecosystem (3000–5000 m above sea level, a.s.l.) is commonly found; however, rain gauge deployment remains logistically demanding due to budgetary constraints, extreme weather conditions, and limited telecommunication coverage in remote and hard-to-access areas, all of which hinder the installation, data retrieval, and long-term maintenance of monitoring stations. Alternative approaches have been explored in similar data-scarce contexts, including regionalization techniques, hydrological indices derived from remote sensing, and transferring flow metrics from nearby gauged basins; however, their applicability in small, high-Andean headwater catchments remains limited by spatial heterogeneity and data availability.

Tropical Andean streams provide an ideal setting to explore ecohydrological trends (such as the role of flow as a regulatory factor of biological communities) as they exhibit strong hydrological seasonality, steep gradients, and exceptional biological diversity [13,14,15], but remain poorly instrumented and monitored. Given this, and acknowledging that “the optimal is the enemy of the good”, a practical alternative for estimating flow is to use rainfall-runoff modeling forced by satellite-derived rainfall [16]. These estimations are generated by combining microwave and infrared observations with atmospheric models, providing spatially continuous precipitation data on a global scale [17]. The main limitations of these products are their coarse resolution and potential bias in tropical regions [18,19]. Nevertheless, numerous studies have validated their use [20,21,22,23].

For Ecuador, satellite-derived rainfall was validated using the National Institute of Meteorology and Hydrology (INAMHI) rain gauge database, given its similarity to surface station observations and its potential for hydrometeorological applications where gauge networks remain sparse [24]. Other studies have also shown good agreement between satellite-derived products and global precipitation datasets [25,26]. Beyond satellite-derived rainfall products, previous studies have explored the use of large-scale atmospheric datasets that indirectly incorporate satellite information through data assimilation. Such datasets have been applied in rainfall–runoff modeling frameworks, showing satisfactory performance in several poorly gauged regions [27,28]. For Ecuador, very limited studies have been conducted using these large-scale precipitation datasets, which may provide a physically consistent representation of the atmospheric system. Related studies have demonstrated that such products exhibit reasonable skill in realistically simulating climate variability over continental Ecuador [29].

Here, we investigated how antecedent flow regimes, derived from rainfall–runoff modeling based on precipitation data indirectly informed by satellite observations, affect the taxonomic and functional structure of benthic macroinvertebrate communities. Specifically, we address the research question of which temporal window, expressed as the number of preceding flow days for a given day of interest, exerts the most decisive influence on macroinvertebrate assemblages. We hypothesize that such antecedent conditions, often described as part of a system’s hydrological memory, capture the cumulative flow dynamics that shape community structure over short temporal scales. To test this hypothesis, we implemented a hydroinformatics protocol that integrates rainfall–runoff modeling with statistical evaluation across multiple antecedent windows representing the days preceding macroinvertebrate sampling. To the best of our knowledge, this study introduces a novel hydroinformatics perspective with the potential of enhancing the quantitative assessment of ecohydrological linkages, highlighting both the scientific robustness and the practical applicability of this framework for data-scarce tropical mountain basins. The concept of hydrological memory is used strictly as a modeling construct rather than as a direct representation of biological or organismal memory. Here, hydrological memory refers to the persistence of antecedent flow conditions, summarized through time-integrated discharge descriptors over predefined temporal windows. These descriptors characterize the recent hydrological context experienced by the stream but do not imply explicit memory mechanisms at the level of individual organisms or communities. Observed biological patterns are interpreted as statistical associations between macroinvertebrate community structure and antecedent hydrological conditions, rather than as evidence of causal ecological memory processes. We further acknowledge that antecedent flow metrics may partly reflect accumulated and correlated uncertainties inherent to rainfall–runoff modeling in data-scarce environments.

## 2. Materials and Methods

### 2.1. Study Area

**Figure 1:**
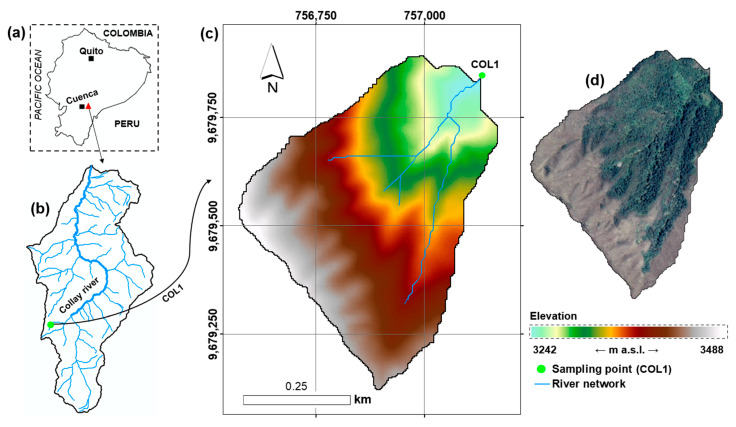
(**a**) Location of the Collay River catchment within continental Ecuador, showing the cities of Quito and Cuenca. (**b**) Hydrographic network of the Collay River catchment and position of the study micro-catchment. (**c**) Stream network and digital elevation model of the micro-catchment with outlet in the bio-sampling point COL1. (**d**) High-resolution satellite image of the study micro-catchment showing land coverage: the green-toned area corresponds to the evergreen montane forest of the southern Eastern Andean Cordillera, whereas the brown-toned area represents the Páramo herbaceous vegetation. Coordinate reference system: WGS 84/UTM Zone 17 S (m).

The study was conducted in a micro-catchment in the upper part of the Collay catchment in southern Ecuador (Figure 1a). This micro-catchment represents the hydrological contributing area to a benthic macroinvertebrate sampling site (COL1) and extents 0.26 km^2^, with a perimeter of 2.76 km. The hydrographic context of the Collay River catchment and the location of the study micro-catchment within it are shown in Figure 1b. The altitudinal range extends from 3242 to 3488 m a.s.l., with a mean slope of 50.1%, as derived from the digital elevation model (Figure 1c). The sampling site and its contributing area are within a remote high-Andean Páramo ecosystem characterized by the absence of direct anthropogenic land-use pressures. While natural environmental variability (e.g., seasonal hydrological fluctuations, frost events, or short-term droughts) is inherent to dynamic mountain systems, no evidence of sustained human disturbance or land-cover change was observed during the study period. This setting allows observed biological patterns to be interpreted primarily in relation to hydrological variability rather than to confounding anthropogenic impacts. In contrast, other micro-catchments with some degree of anthropogenic pressure could introduce misleading effects, as the variability in macroinvertebrate assemblages might reflect external disturbances rather than hydrological processes. The micro-catchment was delineated using Geographic Information Systems (GIS) tools based on a high-resolution digital elevation model (DEM) derived from a LiDAR dataset produced by the SIGTIERRAS project of the Ecuadorian government [30]. The DEM has an original horizontal resolution of 3 m and a vertical precision of ±1.5 m. Flow-direction and flow-accumulation grids were used to identify the contributing area upstream of the COL1 bio-sampling point, defining the specific hydrological unit analyzed in this study (Figure 1c) [31]. A high-resolution satellite image is shown in Figure 1d, illustrating the dominant land-cover types, including evergreen montane forest and herbaceous Páramo vegetation. Because of the site’s remoteness, in situ weather data are unavailable for the evaluated micro-catchment. However, the prevailing climatic conditions are consistent with those expected for high-Andean Páramo ecosystems, characterized by air temperatures ranging from approximately 6 to 17 °C and monthly accumulated precipitation ranging from approximately 30 to 700 mm [32].

### 2.2. Sampling of Benthic Macroinvertebrates

Benthic macroinvertebrates were collected from the study’s (unnamed) tributary of the Collay River. Sampling was conducted within an approximately 10 m stream reach using a 25 × 25 cm^2^ hand-net with a 0.5 mm mesh. The substratum immediately upstream of the net was disturbed by foot to dislodge benthic fauna, following a standardized kick-sampling procedure. Each sampling event comprised a 3 min active search covering all available microhabitats and substrate types [33]. Twenty temporal replicates were collected between 2008 and 2017, including one sample from 2008, two from 2017, and the rest distributed across the intermediate years (2010–2013). All specimens were preserved in 70% ethanol and later sorted out and identified to the family level using a stereomicroscope.

### 2.3. Calculation of Benthic Macroinvertebrate Taxonomic Diversity Indices

Macroinvertebrate community data from the 20 temporal replicates were used to compute both taxonomic and functional diversity metrics, representing complementary aspects of community structure. Taxonomic diversity was described using three classical indices [34]: (i) Shannon diversity (H): integrating richness and evenness; (ii) Pielou evenness (E): expressing the uniformity of relative abundances; and (iii) Simpson dominance (D): representing community concentration.

### 2.4. Calculation of Benthic Macroinvertebrate Functional Diversity Indices

To calculate the functional diversity (FD) indices, eight biological traits were selected (Table 1). They encapsulate 41 functional macroinvertebrate categories (FMaC). The selected traits and their categories are well-known indicators of ecohydrological stress [35,36]. A trait database at the family level (Db_f-trait_) was constructed for the sampling point and its replicates. Each family was assigned affinity scores using a fuzzy coding procedure [37] based on the information gathered from Sotomayor et al. [38] for the Paute River basin, which encompasses the micro-catchment evaluated in this study. The affinity scores varied in the interval [0, 3]; a score value of 0 depicts no affinity of the taxon for the trait category, and a score of 3 shows total affinity. The values of the hardness of the exoskeleton (see Table 1) were assigned based on the expertise of the authors. Functional diversity indices were then computed from the resulting Db_f-trait_ matrix. The following indices were derived from this matrix: Rao’s quadratic entropy (Rao) [39]—capturing overall trait dispersion within the community; Functional Attribute Diversity (FAD1) [40]—reflecting total trait dissimilarity among taxa; and Community-weighted functional divergence (wFDc) [41]—emphasizing dominance and niche differentiation in trait space.

### 2.5. Rainfall Data and Rainfall–Runoff Modeling

Daily satellite-derived rainfall (SDR) was used as input for rainfall–runoff modeling. The model was configured for the study micro-catchment, which was represented as a single hydrological unit directly connected to the micro-catchment outlet. This aggregated modeling approach is assumed to be valid in this study, given the minimal spatial extent of the study site (i.e., 0.26 km^2^) [42]. Rainfall–runoff was modeled using the Soil Conservation Service Curve Number (SCS-CN) method [43,44] under Antecedent Moisture Condition III (AMC III) [45]. Land cover within the micro-catchment was represented, through the QGIS Global Curve Number workflow, by two dominant classes representative of high-Andean Páramo ecosystems: herbaceous vegetation and forest, the latter characterized by scattered montane tree species such as *Polylepis* sp. (Figure 1d). Curve Number values were assigned following standard SCS guidelines based on land-cover class and soil hydrologic group, as implemented within the GIS workflow described in the referenced method [43,44,45], resulting in an overall Curve Number of 86.54 for the study micro-catchment, which was used as input for the rainfall–runoff simulations. The QGIS Global Curve Number tool implements the SCS-CN method by integrating global, high-resolution land-cover and soil datasets (ESA WorldCover 2021 and ORNL HYSOG), and computes a spatially distributed CN raster by intersecting land-cover classes and soil groups under user-defined hydrologic and antecedent runoff condition settings. Under Antecedent Runoff Condition III, representing wet-season conditions prevailing during the biological sampling periods, the resulting basin-average CN reflects high antecedent soil moisture rather than land-use degradation or anthropogenic disturbance. This parameterization is therefore intended to represent hydrologically wet conditions at the time of sampling, which occurred predominantly during the wet season (October–March), rather than long-term average conditions of the catchment. Flow routing was performed using the Clark Unit Hydrograph (CUH) method [46], with a time of concentration of 24 h and a storage coefficient of 12 h, ensuring numerical stability at a daily time step. Baseflow was simulated with a recession model, using an initial discharge of 0.005 m^3^ s^−1^ and a recession constant of 0.95 day^−1^, representing sustained but minimal flow. Daily simulations were executed for all the years corresponding to macroinvertebrate sampling campaigns (2008, 2010, 2011, 2012, 2013, and 2017). The resulting modeled discharge time series (Q_sim_, m^3^ s^−1^) was used for the subsequent calculation of antecedent flow indices. Direct validation of Q_sim_ was not feasible because of the absence of streamflow gauging stations within or near the study catchment, a common limitation in remote, high-altitude headwater systems. Conventional calibration or validation against observed discharges was not possible. To provide an independent assessment of the credibility of SDR for future ecohydrological applications in the study site, besides a direct comparison of the SDR and ground-gauged rainfall, a consistency exercise was conducted to evaluate whether discharges generated with SDR are comparable to those obtained using ground-based rainfall. Specifically, ground-based daily precipitation records from a nearby INAMHI meteorological station located approximately 10 km from the study site were used in the same rainfall–runoff modeling framework that was used with the SDR to generate the discharge estimates considered in this study. Because of temporal gaps in the ground-based rainfall records, the comparison was restricted to continuous periods present in both precipitation datasets. All analyses were further limited to years corresponding to macroinvertebrate sampling campaigns, ensuring temporal coherence between hydrological forcing and biological response. A concise summary of this consistency assessment is provided in the Supplementary Material, which also presents methodological details and comparative simulation results. Overall, discharge simulations driven by SDR and ground-based rainfall exhibited comparable temporal behavior during the shared continuous periods, supporting the use of SDR to reconstruct antecedent flow conditions relevant for ecohydrological analysis.

### 2.6. Calculation of Antecedent Flow Metrics

From the simulated daily discharge series, some hydrological descriptors were computed to characterize the short-term flow history prior to each macroinvertebrate sampling event. Three temporal windows, selected to balance ecological significance against hydrological uncertainty, were defined to represent different antecedent conditions: Q3, Q6, and Q9, corresponding respectively to the mean discharge conditions during the 3-, 6-, and 9-day periods preceding the sampling day. Within each window, hydrological variability was summarized through a set of indices describing both flow magnitude and temporal variability, including: (i) mean discharge (Q_mean_) [47]; (ii) Richards–Baker flashiness index (R–B) [1]; (iii) frequency of positive flow changes (nΔQ > 0); (iv) sum of positive flow changes (ΣΔQ > 0); sum of negative flow changes (ΣΔQ < 0) [48,49,50]; and (v) total absolute flow change (Σ|ΔQ|) (Table 2). These indices capture complementary aspects of the short-term hydrological regime, integrating magnitude, variability, and flashiness. The resulting Q3, Q6, and Q9 datasets were used as quantitative predictors (using their hydrological descriptors) in subsequent analyses linking antecedent flow dynamics to macroinvertebrate diversity.

### 2.7. Rationale for Indices Selection

The rationale behind the selection of the biological and hydrological indices used in this study was to integrate complementary perspectives on community organization and hydrological forcing, providing a coherent basis for assessing ecohydrological relationships. The approach combines indices that differ in how they quantify biological structure and flow variability, ensuring both ecological interpretability and methodological robustness. For functional diversity, the following indices were selected: Rao, FAD1, and wFDc. Together, they capture different dimensions of functional organization (from overall trait dispersion and redundancy to community-weighted differentiation) thus encompassing the diversity of ecological strategies within assemblages. For taxonomic diversity, three classical indices were considered: H, E, and D, which jointly summarize richness–evenness structure and community dominance. These biological metrics collectively describe both the compositional and functional facets of diversity, allowing an integrated interpretation of macroinvertebrate community patterns. In this context, some authors say species identification is needed for diversity metrics like the ones used here [34]. However, in agreement with related studies [51,52,53,54,55], Sotomayor et al. [38] demonstrated that diversity indices based on benthic macroinvertebrate families are useful for stream bioassessment in the Paute River Basin.

The hydrological metrics (i.e., Q_mean_, R–B, nΔQ > 0, ΣΔQ > 0, ΣΔQ < 0, and Σ|ΔQ|) were selected to represent the principal dimensions of short-term antecedent flow: magnitude, variability, and directionality. This set captures the balance between hydraulic stability and disturbance, which is central to understanding flow-related ecological responses in streams. A specific consideration concerns the choice of the temporal windows (3-, 6-, and 9-day) used to quantify antecedent flow. While these windows may appear somewhat arbitrary, they were selected to balance ecological relevance with hydrological uncertainty. Uncertainties accumulate progressively in rainfall–runoff modeling as antecedent periods lengthen, particularly in remote mountain environments [56,57]. In this sense, extending the window further would compound errors, analogous to weather forecasting, where predictive skill rapidly declines beyond short temporal horizons [58]. The 3-, 6-, and 9-day periods therefore represent a pragmatic compromise: they are sufficiently long to summarize recent hydrological history, yet short enough to limit the propagation of modeling uncertainty associated with extended antecedent periods. Rather than aiming to identify a single ‘optimal’ antecedent window, our aim was to evaluate whether ecologically meaningful responses emerged consistently across short-term temporal scales. This approach avoids overfitting window selection to a limited dataset and emphasizes robustness and interpretability over exhaustive optimization. Overall, the combined use of taxonomic, functional, and hydrological indices provides a multidimensional framework that links biological organization to the temporal dynamics of flow. This design ensures that observed relationships reflect ecologically meaningful mechanisms rather than statistical coincidence, providing a robust basis for evaluating how antecedent flow affects benthic macroinvertebrate diversity. In the subsequent analytical stage, biological diversity indices were evaluated as response variables in relation to the hydrological predictors, providing an explicit test of these ecohydrological linkages.

### 2.8. Data Analysis

An initial exploratory analysis was conducted to characterize the relationships between hydrological descriptors and biological indices across the three antecedent windows (Q3, Q6, and Q9). For each window, hydrological and biological variables were jointly displayed in faceted plots to facilitate visual assessment of their temporal co-variation. Because these variables differ in magnitude, all indices plotted on the *y*-axis were transformed via range scaling to the interval [0, 1] [59]. This transformation was applied only for visualization, enabling direct comparison of patterns while preserving the relative dynamics of each variable; all subsequent analyses were conducted on the original, unscaled data. To complement the visual inspection and formally quantify the strength and direction of the associations, a Spearman rank correlation coefficient (ρ) was computed for each hydrological–biological pair within every antecedent window. Spearman’s ρ was selected because it does not assume linearity, is robust to small sample sizes, and is appropriate for detecting monotonic ecological responses [60].

Given the limited number of biological observations (*n* = 20) and their uneven temporal distribution across years, the present analysis does not aim to reconstruct continuous temporal trajectories or seasonal dynamics of macroinvertebrate communities. Instead, each sampling event is treated as a discrete temporal snapshot, interpreted in relation to the short-term antecedent hydrological context preceding the observation. Associations between hydrological descriptors and biological indices are evaluated at the level of individual sampling events rather than as evidence of long-term trends. Long temporal gaps between sampling years may partially confound hydrological effects with broader climatic or ecological variability; therefore, results are interpreted as localized ecohydrological responses rather than as evidence of long-term trends.

Following the exploratory assessment, the analytical framework examined how prior water conditions influenced changes in benthic macroinvertebrate diversity. Each biological index—encompassing both taxonomic (H, E, D) and functional (Rao, FAD1, wFDc) indices—was treated as a response variable, whereas the set of hydrological descriptors (Q_mean_, Richards–Baker index, nΔQ > 0, ΣΔQ > 0, ΣΔQ < 0, and Σ|ΔQ|) represented the predictor block. Analyses were performed independently for each antecedent window (Q3, Q6, and Q9) to determine the temporal scale at which flow variability had the most decisive influence on biological responses. Generalized Additive Models (GAMs) were fitted to explore potential non-linear relationships between biological and hydrological indices [61,62]. To account for potential temporal dependence among sampling events, models were first fitted using a first-order autoregressive correlation structure (AR(1)) based on the event index. When this specification failed to converge, a standard GAMs including a smooth term over the event index was adopted as a fallback strategy. Given the limited number of observations and the quasi-linear nature of most relationships, smooth functions were constrained to low degrees of freedom (basis dimension k = 3) or effectively reduced to linear terms when penalization yielded effective degrees of freedom close to one. This ensured model parsimony while retaining sufficient flexibility to capture moderate nonlinearity in the data [63]. To assess model reliability, three complementary criteria were considered: (i) explanatory power, expressed as the adjusted coefficient of determination (R^2^_adj_) [64]; (ii) prediction accuracy, expressed as the root-mean-square error (RMSE) [65]; and (iii) the distribution of residuals, evaluated using the Shapiro–Wilk test for normality (*p* ≥ 0.05 showing normal residuals) [66]. Models meeting these three criteria (high R^2^_adj_, low RMSE, and approximately normal residuals) were considered the most reliable and were prioritized for subsequent interpretation. Although some models used a non-Gaussian family (e.g., beta regression for proportion-type indices), the Shapiro–Wilk test was applied as a general diagnostic of residual behavior rather than as a strict test of distributional assumptions. For models fitted with a Gaussian error distribution, residual normality is an appropriate adequacy check; for non-Gaussian models, it was used descriptively to ensure that no extreme deviations affected model interpretability [62].

#### Assessing the Most Significant Hydrological Descriptive Parameters

Identification of the most informative hydrological descriptors was performed only for the best-performing antecedent window, previously selected according to the highest R^2^_adj_, the lowest RMSE, and residual normality confirmed by the Shapiro–Wilk test (Section 2.6). For this optimal model, the relative contribution of each hydrological variable was evaluated using a drop-one approach based solely on the change in adjusted R^2^ (ΔR^2^_adj_). Each predictor was sequentially excluded from the model, and the resulting change in R^2^_adj_ was computed to quantify its influence on the explained biological variability. Greater reductions in R^2^_adj_ showed higher predictor importance, whereas negligible changes suggested limited contribution [67,68]. ΔR^2^_adj_ values were interpreted comparatively to identify the most informative descriptors. This procedure provided a transparent and robust basis for ranking hydrological parameters [69]. The resulting ranking thus reflects the descriptors that most strongly shaped the biological responses under the hydrological integration period identified as most representative of the system’s ecohydrological memory.

### 2.9. Hydrological Code and Auxiliary Software

The contribution area above the bio-sampling site (COL1) was delineated in ArcGIS v. 10.8.2 [31] using the flow-direction and accumulation method [70], based on digital elevation data. Functional and taxonomic diversity indices were computed using FDiversity software [71] (v. 2008). Daily precipitation was obtained from the NASA Prediction of Worldwide Energy Resources (POWER) [72] database and used as input for rainfall–runoff modeling with the Hydrologic Engineering Centre’s Hydrologic Modeling System, HEC-HMS v. 4.12 [73]. Curve Numbers required for the rainfall–runoff model were derived through a GIS-based workflow implemented in QGIS, using the Global Curve Number tool, which integrates land-cover and soil hydrologic group information following standard SCS guidelines. Official land-cover datasets from the Government of Ecuador [74] were additionally consulted for contextual comparison and visual verification of land-cover patterns at the study site. This comparison indicated general agreement with the land-cover representation got through the QGIS workflow, particularly regarding the spatial distribution of Páramo herbaceous vegetation and forested areas. All subsequent hydrological and statistical analyses were conducted in R v. 4.3.2. Hydrological descriptors were computed using the packages tidyverse [75] (v. 2.0.0), lubridate [76] (v. 1.9.4), and hydrostats [77] (v. 0.2.9). The exploratory visual analysis of hydrological–biological associations (which involved rescaling variables to the [0, 1] interval and computing Spearman rank correlation coefficients) was implemented using functions from tidyverse and base R. Following this exploratory step, GAMs were fitted to evaluate hydrological–biological relationships using the mgcv [78] (v. 1.9.1) and nlme [63] (v. 3.1.166) packages.

## 3. Results

### 3.1. Hydrological Modeling and Flow Characterization

Daily rainfall from the NASA POWER dataset was used to simulate discharge in the study micro-catchment using HEC-HMS. The simulation reproduced a dynamic hydrological regime typical of small Andean headwaters, with daily precipitation ranging from approximately 0 to 40 mm day^−1^ (mean ≈ 3.1 mm day^−1^) and corresponding simulated flows ranging from 0.0005 m^3^ s^−1^ under baseflow conditions to about 0.10 m^3^ s^−1^ during rainfall events (mean ≈ 0.01 m^3^ s^−1^). The coefficient of variation for simulated discharge exceeded 100%, showing pronounced short-term hydrological variability throughout the study period. For context, a small set of spot discharge measurements collected during field visits at the sampling point (COL1) spanned the same order of magnitude as simulated flows (approximately 0.003–0.14 m^3^ s^−1^). These observations were not used for calibration or formal validation because of their limited number and incomplete temporal coverage, but they support the plausibility of the simulated discharge range. Although direct calibration or validation was not feasible because of the limited number of field records, the simulated hydrographs exhibited plausible temporal responses to rainfall. They seemed to provide a consistent basis for deriving descriptive hydrological parameters.

### 3.2. Exploratory Co-Variation Across Temporal Windows (Q3, Q6, Q9)

**Figure 2:**
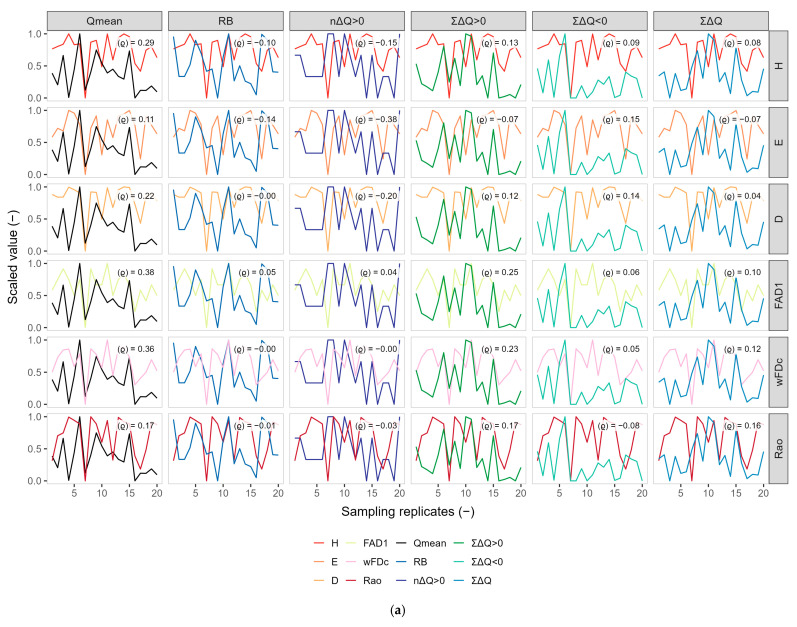

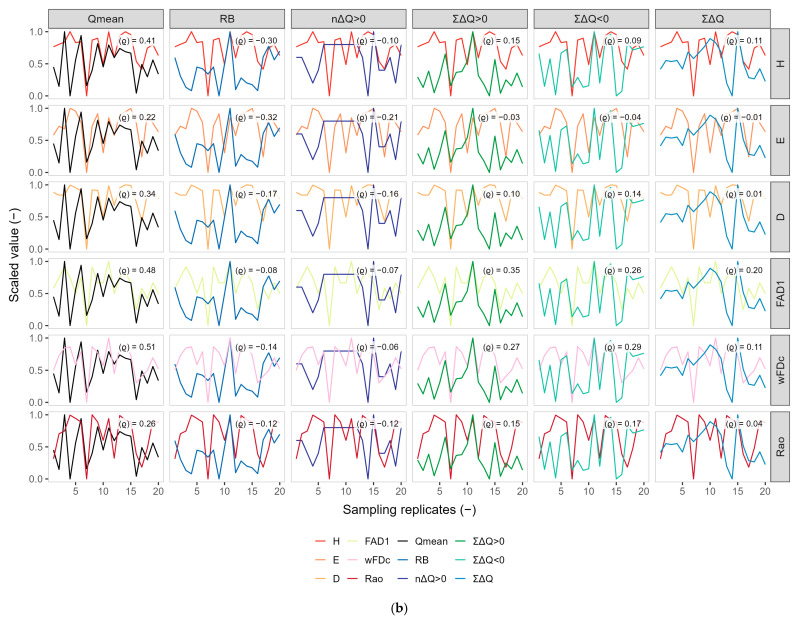

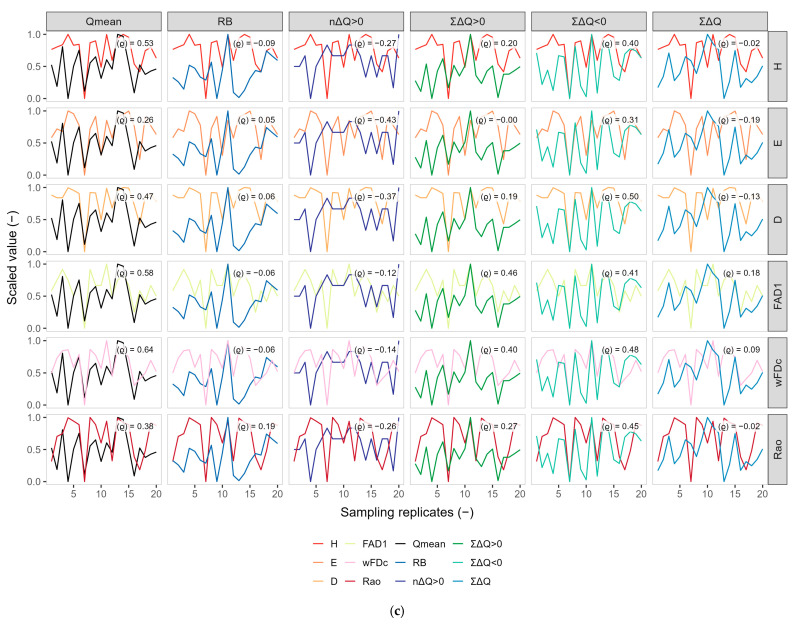
Temporal co-variation between hydrological descriptors and macroinvertebrate diversity indices across three antecedent-flow windows (**a** = Q3, **b** = Q6, **c** = Q9). For each window, standardized temporal profiles (0–1 scale) are shown for hydrological and biological variables. Each panel includes the corresponding Spearman correlation coefficient (ρ) summarizing the strength of association between paired hydrological and biological descriptors. Hydrological indices: Q_mean_ = mean daily discharge; RB = Richards–Baker flashiness index; nΔQ > 0 = frequency of positive flow changes; ΣΔQ > 0 = sum of positive flow changes; ΣΔQ < 0 = sum of negative flow changes; Σ|ΔQ| = total absolute flow change. Biological indices: H = Shannon diversity; E = Pielou evenness; D = Simpson dominance; FAD1 = pairwise trait-based dispersion; wFDc = centroid-weighted functional distance; Rao = Rao’s quadratic entropy.

The strength and direction of the pairwise associations between hydrological descriptors and biological metrics varied systematically across the antecedent windows (Q3, Q6, Q9) (Figure 2), revealing a clear temporal hierarchy in hydrological–biological coupling. Under the 3-day window, most correlations were weak to moderate, showing limited integration of very short-term flow variability. Nevertheless, mean discharge (Q_mean_) already showed modest positive associations with both taxonomic and functional indices (e.g., FAD1: ρ ≈ 0.38; wFDc: ρ ≈ 0.36), suggesting that baseline flow conditions may exert an early integrative influence on community structure. Cumulative change metrics (ΣΔQ > 0 and ΣΔQ < 0) exhibited only weak and inconsistent relationships at this temporal scale, while descriptors of flashiness and event frequency (R–B and nΔQ > 0) remained uncorrelated with biological metrics. Within the 6-day window, correlation patterns became more structured, particularly for descriptors representing accumulated hydrological conditions. Q_mean_ displayed stronger positive associations with diversity indices (e.g., wFDc: ρ ≈ 0.51), while ΣΔQ > 0 and ΣΔQ < 0 showed moderate positive correlations with functional metrics (FAD1: ρ = 0.35 and 0.26; wFDc: ρ = 0.27 and 0.29, respectively). In contrast, R–B and nΔQ > 0 continued to exhibit weak or predominantly negative relationships, indicating that short-term variability and event frequency alone were insufficient to explain biological responses. The 9-day window revealed the clearest and strongest patterns across the entire analysis. Both baseline and cumulative descriptors reached moderate to high correlation magnitudes, highlighting the importance of integrated hydrological context. Q_mean_ showed consistently high positive correlations with functional diversity (e.g., wFDc: ρ ≈ 0.64), while ΣΔQ < 0 exhibited the highest Spearman coefficients overall (FAD1: ρ = 0.41; wFDc: ρ = 0.48; Rao: ρ = 0.45; D: ρ = 0.50), showing that sustained decreases in discharge exert coherent influences on functional structure. ΣΔQ > 0 also displayed stable positive associations with functional metrics (FAD1: ρ = 0.46; wFDc: ρ = 0.40). Frequency-based descriptors (nΔQ > 0) remained negatively associated with most biological indices (e.g., H: ρ = −0.27; E: ρ = −0.43), reinforcing the interpretation of high-frequency variability as a short-term disturbance signal. Overall, the progressive increase in correlation strength from Q3 to Q9 shows that macroinvertebrate communities respond primarily to hydrological conditions accumulated over several days rather than to isolated daily events.

### 3.3. Generalized Additive Models (GAMs)

**Figure 3:**
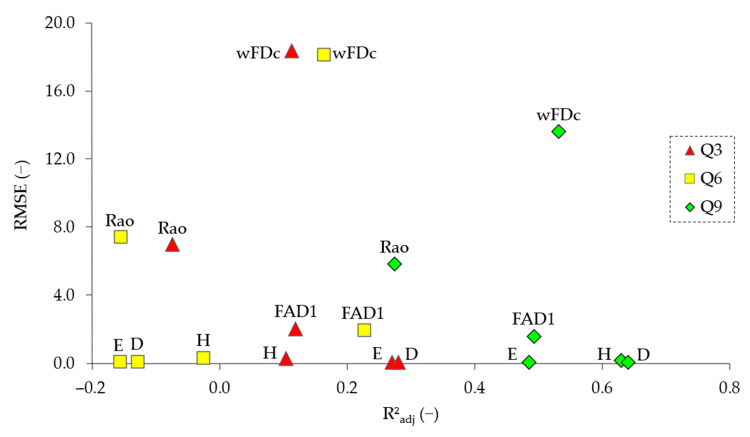
Comparative visualization of generalized additive model (GAM) performance indices for each biological response variable across the three antecedent-flow windows (Q3, Q6, and Q9). The figure reports the adjusted coefficient of determination (R^2^_adj_) and the root-mean-square error (RMSE) obtained for each model. Biological indices include H (Shannon diversity index), E (Pielou evenness), D (Simpson dominance), FAD1 (functional attribute diversity index), wFDc (community-weighted functional dispersion based on continuous traits), and Rao (Rao’s quadratic entropy).

All reported metrics (R^2^_adj_, RMSE) come from GAMs fitted separately for each antecedent-flow window (Q3, Q6, Q9). No mixed/correlation structure was retained; therefore, the results pertain only to GAM fits. Across all biological responses (H, E, D, FAD1, wFDc, Rao), model performance improved consistently with increasing antecedent-window length, with Q9 outperforming both Q6 and Q3 (Figure 3). According to the model summaries, Q9 achieved the highest overall explanatory power (mean R^2^_adj_ = 0.51 ± 0.12, with all six responses showing R^2^_adj_ ≥ 0.20) and the lowest mean prediction error (RMSE = 3.58 ± 4.94). In contrast, Q3 showed modest performance (mean R^2^_adj_ = 0.14 ± 0.12; with 2/6 responses ≥ 0.20) with a RMSE = 4.70 ± 6.60, whereas Q6 performed poorly (mean R^2^_adj_ = −0.01 ± 0.15; with 1/6 responses ≥ 0.20) and exhibited a higher average RMSE (4.70 ± 6.54). Individually, each response selected Q9 as the best-performing window by R^2^_adj_: H (0.630), E (0.485), D (0.640), FAD1 (0.493), wFDc (0.532), and Rao (0.275). This consistent improvement with increasing window length suggests that benthic macroinvertebrate diversity integrates hydrological variability over approximately a week to ten days.

Among the models fitted for the best-performing window (Q9), the residuals of five out of six biological indices (H, E, FAD1, wFDc, and Rao) followed an approximately normal distribution (*p* ≥ 0.05), confirming their statistical validity under the GAM assumptions. In contrast, the model for Simpson dominance (D) exhibited a significant departure from normality (*p* = 0.03 < α = 0.05), suggesting a potential mild violation of residual assumptions. Although this deviation does not invalidate the D model, it shows reduced robustness relative to the other biological indices. Therefore, the D model was interpreted with caution and was not prioritized in subsequent comparative analyzes. Overall, the residual inspection supports the adequacy of the GAMs approach for representing the relationship between antecedent flow variability and macroinvertebrate diversity, with only minor exceptions.

### 3.4. Comparative Performance of Taxonomic and Functional Responses Under the Best Temporal Window (Q9)

Within the best-performing window (Q9), taxonomic indices (H, E, D) exhibited stronger and more consistent relationships with antecedent hydrological conditions than functional metrics (FAD1, wFDc, Rao). The GAMs for taxonomic responses achieved higher explanatory power (mean adjusted R^2^_adj_ = 0.59 ± 0.08) compared with functional responses (mean adjusted R^2^_adj_ = 0.43 ± 0.11). Prediction errors followed the same pattern, with lower RMSE values for taxonomic metrics (mean RMSE = 0.11 ± 0.06) relative to functional metrics (mean RMSE = 6.37 ± 5.84). Among the taxonomic variables, H (Shannon diversity) and D (Simpson dominance) displayed the strongest fits (R^2^_adj_ = 0.63–0.64), whereas E (evenness) achieved slightly lower but still robust performance (R^2^_adj_ = 0.48). Within the functional group, wFDc and FAD1 showed intermediate explanatory capacity (R^2^_adj_ = 0.53 and 0.49, respectively), while Rao presented the weakest response (R^2^_adj_ = 0.27). These results show that, under the same hydrological forcing, taxonomic metrics captured a greater proportion of the biological variability than functional ones, suggesting that compositional changes in macroinvertebrate assemblages are more directly responsive to antecedent flow variability than changes in trait-based structure.

### 3.5. Hydrological Parameters Under the Best-Performing Window (Q9)

Under the nine-day antecedent window (Q9), the relative influence of each hydrological parameter on the five evaluated biological indices (H, E, FAD1, wFDc, Rao) was examined using changes in R^2^_adj_ (ΔR^2^_adj_) after the sequential exclusion of each variable. Parameters with larger ΔR^2^_adj_ values were considered more informative for explaining biological variability. Table 3 summarizes the ΔR^2^_adj_ results for each response variable under Q9. Across indices, the sum of negative flow changes (ΣΔQ < 0) showed the highest explanatory relevance. This parameter captures the combined effects of hydrological stability (recession) and short-term variability (flashiness) on benthic community structure. The model for D (Simpson dominance) was excluded from this analysis because of non-normal residuals.

The magnitude of ΔR^2^_adj_ values was clearly higher for the taxonomic indices (H, E) than for the functional ones (FAD1, wFDc, Rao). This confirms that hydrological parameters exerted a stronger and more consistent influence on taxonomic structure than on functional diversity. The pattern is fully congruent with the preceding results based on overall model fit (R^2^_adj_, RMSE) and residual diagnostics, which also showed superior performance for the taxonomic models. Together, these findings show that compositional attributes of macroinvertebrate assemblages are more sensitive to antecedent hydrological variability, whereas trait-based metrics display weaker and less consistent responses under the same environmental forcing.

## 4. Discussion

### 4.1. Using Satellite Rainfall Data with Hydrological Modeling

Runoff estimation in ungauged catchments remains a challenge in hydrology, highlighted by initiatives such as Prediction in Ungauged Basins [79]. In remote tropical Andean basins, logistical and financial constraints often limit the installation and maintenance of ground-based meteorological networks, despite the key regulatory role of the Páramo ecosystem [80,81]. Within this context, satellite-derived rainfall (SDR) datasets have been used in several studies, as well as large-scale precipitation products that assimilate satellite information and provide climatic consistency; this may explain their close agreement with observed data, as shown by comparisons with a nearby surface station evaluated in the present study (see Supplementary Material). In this study, we adopted this general framework and performed rainfall–runoff modeling using a global precipitation dataset, an application that has shown good performance in previous studies [82,83,84]. Specifically, we employed the NASA POWER rainfall product, which several studies have reported to deliver acceptable performance for hydrological applications across diverse settings [20,21,22,23]. Building on this evidence, we applied the aggregated HEC-HMS code with daily NASA POWER rainfall to estimate discharge dynamics in the Collay micro-catchment. The simulated hydrographs reproduced persistent baseflow and plausible peak variability at daily resolution, providing an estimated hydrological series from which multi-day antecedent flow descriptors (Q3, Q6, Q9) were derived. Although observed streamflow data were not available for formal calibration, plausibility checks based on the runoff coefficient (ratio of simulated discharge Q to precipitation P) and the stability of baseflow recession constants supported the reliability of the simulations as a sound ecohydrological basis.

### 4.2. Optimal Antecedent Window and Ecological Interpretation (Q9)

Model comparisons among the three antecedent windows (Q3, Q6, Q9) revealed a consistent improvement in model performance with increasing temporal integration, identifying Q9 as the optimal timescale for explaining biological variability. Shorter windows (e.g., Q3) capture highly transient hydrological fluctuations that do not translate into biological change, whereas the nine-day antecedent flow integrates the dominant environmental conditions that structured the community before sampling. From a hydrological perspective, short antecedent periods mainly reflect isolated disturbances (such as single rainfall events or temporary low-flow episodes) that dissipate rapidly and therefore have limited ecological persistence [85]. In contrast, a nine-day window provides a more holistic representation of the flow regime, smoothing daily noise and capturing the cumulative hydraulic stress acting on the streambed. This cumulative signal more accurately reflects the habitat stability or disturbance preceding sampling, characterizing the hydrological context relevant to ecological responses. From an ecological standpoint, macroinvertebrate communities do not respond to hydrological fluctuations instantaneously but to the cumulative environmental conditions experienced over several days [86]. Their taxonomic and functional structures are shaped by the flow conditions experienced over several consecutive days, as recovery and recolonization after moderate disturbances typically require about one week to ten days [87,88,89,90]. The nine-day antecedent period aligns closely with the ecological integration timescale of benthic assemblages in tropical mountain streams—long enough to encompass post-disturbance stabilization, yet short enough to remain sensitive to recent environmental variability—providing quantitative support for the concept of hydrological memory in tropical high-Andean headwater ecosystems. In this context, hydrological memory is interpreted as an emergent property of cumulative antecedent flow descriptors derived from modeled discharge, rather than as a directly measured ecological or biological memory process.

### 4.3. Dominant Hydrological Drivers and Ecological Implications

Within the nine-day antecedent window (Q9), the sum of negative flow changes (ΣΔQ < 0) emerged as the most informative hydrological descriptor, particularly for Shannon diversity (H) and evenness (E) (Table 3). When this variable was removed from the GAMs, the reduction in ΔR^2^_adj_ was substantially higher than for any other predictor, showing that recession dynamics represent the key mechanism linking antecedent hydrology to biological structure. From a hydrological viewpoint, ΣΔQ < 0 quantifies the cumulative magnitude of discharge declines within the antecedent window, effectively summarizing the stabilizing phase of the hydrograph [48]. In tropical high-Andean headwaters, this phase dominates much of the flow regime, reflecting the gradual transition from post-rainfall peaks to sustained baseflow [91,92]. Such recessions encapsulate the hydraulic stability of the system, smoothing short-term noise and defining the physical conditions under which benthic assemblages reorganize. These stable periods promote substrate consolidation, accumulation of fine organic matter, and restoration of low-velocity microhabitats that serve as refuges during fluctuating flows [93].

Ecologically, the dominance of ΣΔQ < 0 shows that macroinvertebrate diversity increases during stable-flow recessions, when physical disturbance decreases, and competitive interactions and recolonization processes can proceed, consistent with earlier reports [94,95]. During these low-flow phases, habitat stability allows greater coexistence among taxa and a more even distribution of abundances, leading to higher values of H and E. Overall, the predominance of ΣΔQ < 0 underscores that low-flow stability is the principal ecohydrological process connecting antecedent hydrology and biological organization within the studied headwater catchment. Sustained recessions act as the integrating signal of hydrological memory, providing the environmental continuity that supports recolonization and community restructuring after disturbance. These findings are consistent with theoretical frameworks emphasizing the complementary roles of disturbance and stability in maintaining diversity [90,96] and reinforce the idea that maximum community organization occurs under intermediate levels of hydrological variability. Similar patterns have been reported in tropical streams, where macroinvertebrate assemblages respond more strongly to the cumulative effects of hydrological disturbance than to the magnitude of individual spates. In the Australian Wet Tropics and Papua New Guinea, recovery was rapid at small spatial scales but slower after whole-stream disturbance, and resilience was shaped by the persistence of hydrological change rather than discrete high-flow events [97]. These observations are consistent with our finding that recession dynamics over multi-day antecedent windows exert a stronger influence on community structure than individual flood peaks.

### 4.4. Taxonomic Versus Functional Sensitivity to Hydrological Variability

The GAMs for taxonomic metrics (H, E, D) achieved substantially higher explanatory power and lower prediction error compared with those for functional indices (FAD1, wFDc, Rao) (Figure 3). The ΔR^2^_adj_ analysis reinforced this pattern, showing that hydrological predictors contributed larger increments to model performance in taxonomic than in functional models (Table 3). This consistent outcome across multiple evaluation criteria suggests that compositional changes in macroinvertebrate assemblages are more immediately influenced by hydrological variability than shifts in trait-based structure, a result congruent with previous observations [98]. Taxonomic turnover can occur rapidly after hydraulic disturbances through drift or short-term habitat changes, whereas functional diversity integrates slower ecological processes such as recolonization, reproduction, and species replacement. An additional explanatory mechanism is trait redundancy, whereby multiple taxa share similar ecological traits, buffering the overall functional structure against transient species losses or colonization [99]. Also, the functional dataset used here (based on family-level trait assignments) may lack the resolution needed to detect subtle shifts in trait composition [100]. These findings align with a growing body of research showing that taxonomic indicators can be as responsive as, or more responsive than, functional ones to specific environmental gradients, including hydrological variability. For instance, Liu et al. [101] found that taxonomic diversity metrics exhibited greater sensitivity to land-use differences than functional complements, suggesting that species identities can still capture key environmental signals overlooked by trait-based measures. Similarly, Saito et al. [102] reported that trait–environment associations in tropical river systems were weaker than taxon–environment relationships, implying that functional traits may integrate broader ecological tolerances that buffer short-term variability. Beyond running waters, comparable patterns have been observed in lentic and terrestrial ecosystems: in shallow lakes, Zhang et al. [103] documented a more pronounced taxonomic homogenization than functional homogenization under nutrient enrichment, while in alpine grasslands, fertilization experiments revealed diverging trends, with species diversity decreasing as functional diversity increased [104]. Collectively, these results support the view that low concordance between taxonomic and functional diversity does not imply ecological irrelevance but reflects complementarity between dimensions of community organization. Taxonomic metrics provide high-resolution detection of compositional shifts, whereas functional metrics reveal the persistence of ecological strategies underpinning resilience and adaptation. This complementarity emphasizes the need for multi-dimensional biodiversity assessments, particularly in ecohydrological contexts where biological structure is linked to hydrological variability.

Interestingly, the strength of the bivariate hydrology–biology associations (Figure 2) did not translate directly into GAM performance. Functional metrics displayed the highest Spearman correlations with specific components of antecedent flow (particularly Q_mean_, ΣΔQ < 0 and ΣΔQ > 0 under the 9-day window), showing sharp responses to cumulative hydrological disturbances. This apparent discrepancy arises because the GAMs integrate multiple hydrological predictors simultaneously and include a temporal smoother that captures low-frequency temporal structure in the taxonomic data. Functional diversity responds strongly to hydrological features, whereas taxonomic diversity reflects a combination of the composite antecedent flow regime and broader temporal trends. These outcomes highlight that functional and taxonomic metrics provide complementary perspectives: functional indices detect specific flow-related disturbances, while taxonomic indices encode more diffuse environmental and temporal signals.

## 5. Conclusions

This study reveals a consistent ecological signal linking short-term antecedent hydrological variability with benthic macroinvertebrate community structure in a pristine Andean headwater. Among the evaluated windows, the 9-day antecedent period (Q9) showed the most coherent associations with both taxonomic and functional diversity metrics, indicating that benthic communities integrate hydrological conditions over several consecutive days. Under these short integration scales, taxonomic indices exhibited higher explanatory power than functional ones.

This study shows that integrating satellite-based rainfall products, conceptual hydrological modeling, and macroinvertebrate diversity analysis offers a pragmatic pathway to investigate ecohydrological dynamics in remote tropical headwaters where ground-based monitoring is absent. Although the NASA POWER precipitation dataset is much coarser than the study micro-catchment scale, its value lies in providing a temporally consistent regional rainfall signal. Our aim was not to reproduce absolute discharge magnitudes but to reconstruct the sequence of wet and dry conditions that shape short-term antecedent flow. For this purpose, POWER data capture the timing of rainfall events with sufficient fidelity to support exploratory hydrological memory analyzes. The lack of in situ precipitation and streamflow measurements represents an inherent limitation of ungauged Andean systems, as localized rainfall variability and high-altitude microclimates cannot be fully resolved by coarse satellite or large-scale precipitation products. Nevertheless, our analysis relies on internally consistent temporal dynamics rather than calibrated discharge values. The comparative behavior of the Q3, Q6, and Q9 windows emerges from relative (not absolute) hydrological variation. In this context, researchers in remote mountain basins must either refrain from ecohydrological assessment due to data scarcity or use the best available remote information to derive preliminary insights. This study adopts the latter approach, not to produce definitive predictions, but to show what can be learned under severe data constraints and to highlight the need for coordinated field monitoring in high-Andean environments.

The identification of a 9-day hydrological memory window further underscores the importance of accounting for temporal integration when linking flow regimes with biological structure. Although functional indices showed the strongest bivariate relationships with specific hydrological descriptors, taxonomic indices achieved higher explanatory power in multivariate models, indicating that taxonomic and functional metrics capture complementary aspects of ecohydrological variability.

Overall, this work provides an exploratory yet valuable first approximation of the effects of antecedent flow on benthic communities in a pristine, ungauged Andean catchment. The framework developed here (combining multi-window hydrological reconstruction, trait-based analysis, and non-linear modeling) offers a foundation for future ecohydrological research in data-limited tropical regions. The applicability of these findings is therefore greatest for small, minimally disturbed tropical headwater systems, and extrapolation to larger, regulated, or heavily impacted catchments should be undertaken with caution. Long-term installation of high-frequency precipitation and streamflow sensors, coupled with expanded sampling across contrasting land-use settings, will allow the refinement and broader evaluation of the hydrological memory patterns observed here.

## Figures and Tables

**Table 1:** Functional traits and categories used in this study.

Trait	Category
Feeding habits	Collector-Filterer (C-Ft)
	Collector-Gatherer (CG)
	Piercers (Pc)
	Predators (Pr)
	Scrapers (Sc)
	Shredders (Sh)
	Parasite (PA)
Respiration	Tegument (Teg)
	Gill
	Plastron (Pla)
	Spiracle (Spi)
Body form	Streamlined (Str)
	Flattened (Flat)
	Cylindrical (Cy)
	Spherical (Sph)
Maximum body size (mm)	<2.5
	2.5–5
	5–10
	10–20
	20–40
	40–80
Body flexibility (°)	None (<10)
	Low (10–45)
	High (>45)
Locomotion	Flier (Fli)
	Surface swimmer (SS)
	Full water swimmer (FWS)
	Crawler (Cra)
	Burrower (Bur)
	Temporarily attached (TA)
Reproduction	Asexual (As)
	Clutches and cemented (CC)
	Clutches and free (CF)
	Clutches in vegetation (CV)
	Clutches and Terrestrial (CT)
	Isolated eggs and clutches (IEC)
	Isolated eggs and free (IEF)
	Ovoviviparity (Ovi)
Hardness exoskeleton	None
	High
	Moderate

**Table 2:** Hydrological descriptors used to characterize antecedent flow conditions across the Q3, Q6, and Q9 temporal windows. The table summarizes the hydrological indices computed from simulated daily discharge series to describe flow magnitude, variability, and flashiness prior to each macroinvertebrate sampling event, including mean discharge, the Richards–Baker flashiness index, frequency and magnitude of positive and negative flow changes, and total absolute flow variation.

Hydrological Descriptor	Acronym Abbreviation	Unit	Description
Mean discharge	Q_mean_	m^3^ s^−1^	Average discharge during the antecedent window, representing overall flow magnitude preceding sampling.
Richards–Baker flashiness index	R–B	(−)	Quantifies short-term flow variability as the ratio between the sum of absolute day-to-day discharge changes and total discharge; higher values show more rapid fluctuations.
Frequency of positive flow changes	nΔQ > 0	(−)	Number of instances where discharge increased from one day to the next within the antecedent window, reflecting the frequency of rising flows.
Sum of positive flow changes	ΣΔQ > 0	m^3^ s^−1^	Cumulative magnitude of all positive (increasing) flow changes; represents total intensity of rising-flow events.
Sum of negative flow changes	ΣΔQ < 0	m^3^ s^−1^	Cumulative magnitude of all negative (decreasing) flow changes; represents total intensity of recessional or declining-flow events.
Total absolute flow change	Σ|ΔQ|	m^3^ s^−1^	

**Table 3:** Relative importance of hydrological parameters under the nine-day antecedent window (Q9), expressed as ΔR^2^_adj_ after removal of each variable from the Generalized Additive Models. Hydrological parameters: Q_mean_ = mean discharge; RB = Richards–Baker flashiness index; nΔQ > 0 = frequency of positive flow changes; ΣΔQ > 0 = sum of positive flow changes; ΣΔQ < 0 = sum of negative flow changes; Σ|ΔQ| = total absolute flow variation. Bold values show the most influential variable per biological index (see the previous table for definitions of biological indices).

Biological Index	Hydrological Parameters					
	Q_mean_	RB	nΔQ > 0	ΣΔQ > 0	ΣΔQ < 0	Σ|ΔQ|
H	0.02	0.04	0.09	−0.01	**0.22**	−0.01
E	0.01	0.09	0.00	0.00	**0.31**	0.00
FAD1	0.01	−0.02	0.08	0.00	0.00	0.00
wFDc	0.02	0.01	−0.06	−0.12	0.00	−0.12
Rao	0.08	0.03	0.02	0.00	0.00	0.01

## Data Availability

All data supporting the findings of this study are provided as Supplementary Material in a single Excel file.

## Supplementary Materials

The following supporting information can be downloaded at: https://www.mdpi.com/article/10.3390/biology15030257/s1. Supplementary Data S1: Excel file containing all datasets and scripts used in this study. The first worksheet includes the complete taxonomic abundance matrix and functional trait matrix for the 20 temporal macroinvertebrate samples collected at site COL1, formatted for direct use in FDiversity software (v. 2008). Subsequent worksheets include (i) precipitation and terrain data obtained from the NASA POWER database for the corresponding sampling years, (ii) simulated daily discharge series generated using HEC-HMS, and (iii) a condensed matrix integrating hydrological descriptors and biological metrics. The final worksheets (to R–Q3, to R–Q6, and to R–Q9) provide the hydrological and biological input matrices used for the generalized additive model (GAM) analyses, ensuring full reproducibility of the modeling workflow. The supplementary file also includes the complete R script used for model fitting and predictor importance evaluation. Additional supplementary tables and figures document the consistency assessment between satellite-based rainfall– and ground-based rainfall–driven discharge simulations described in Section 2.3.
